# Tailoring Enhanced Elasticity of Crystalline Coordination Polymers

**DOI:** 10.1021/acs.cgd.2c01397

**Published:** 2023-02-13

**Authors:** Ozana Mišura, Mateja Pisačić, Mladen Borovina, Marijana Đaković

**Affiliations:** Department of Chemistry, Faculty of Science, University of Zagreb, Horvatovac 102a, Zagreb 10000, Croatia

## Abstract

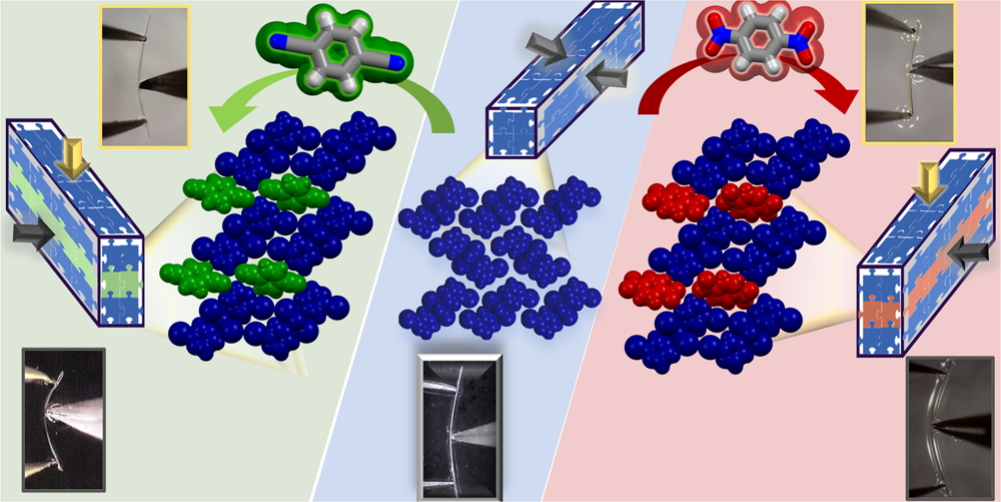

The approach for enhancing the elasticity of crystals with suboptimal elastic performances through a rational design was presented. A hydrogen-bonding link was identified as a critical feature in the structure of the parent material, the Cd(II) coordination polymer [CdI_2_(I-pz)_2_]_*n*_ (I-pz = iodopyrazine), to determine the mechanical output and was modified via cocrystallization. Small organic coformers resembling the initial organic ligand but with readily available hydrogens were selected to improve the identified link, and the extent of strengthening the critical link was in an excellent correlation with the delivered enhancement of elastic flexibility materials.

## Abstract 2

Enhancement of crystal elastic flexibility through a rational design is presented. Introducing a new component into a cadmium(II) CP crystal structure strengthens the hydrogen bonds, identified as a critical feature in determining mechanical responsiveness, whereas the extent of strengthening has an excellent correlation with the delivered enhancement of elastic flexibility materials.

Advanced technologies are continuously imposing additional requirements on materials that are to be implemented in smart devices,^1−3^ and flexibility has a leading role in the toolbox of advanced material properties.^4−6^ Crystalline materials are, due to a long-range order and consequent energy transfer, highly desired,^7,8^ but most of those materials are considered inelastic, and their applicability in advanced technologies is still exceptionally limited.

In addition to the relatively small number of highly elastic and durable crystalline materials reported so far,^9−13^ there are a number of examples that demonstrate crystalline materials could also display suboptimal elastic performances.^14,15^ These materials, although not being extensively elastic, can still adapt to an external mechanical force, displaying slight elasticity prior to cracking, breaking, or disintegrating of their original shape otherwise.

Improving suboptimal properties or performances of high-value substances has been a largely adopted approach, employed for a range of properties and performances, from altering solubility^16^ or modulating hygroscopicity^17^ of active pharmaceutical ingredients (APIs) to improving the mechanical properties of a variety of materials, such as ceramics,^18^ lightweight metal-based materials,^19^ and organic polymers.^20,21^ However, enhancing the mechanical adaptability of crystalline materials to an external mechanical force has not yet been the focus of material research. Herein, we present the first example of elastically flexible crystals whose low elastic performance was successfully improved via strengthening the weakest link in the crystal structure that was identified as critical for the delivery of elastic mechanical output.^9^

In our previous research, we demonstrated that crystals of a family of isostructural materials, coordination polymers (CPs), can display a diversity of elastic extents (presented with different bending strain values, ε), where slight differences in the importance and influence of the intermolecular interactions in the crystal structure, in particular hydrogen bonds, emerged as being critical for the delivery of different elastic performances.^9^ Namely, all the materials consisted of 2-D layers of doubly halogen-bonded 1-D coordination polymers, which were further organized into 3-D assemblies via hydrogen links of different importance: in particular, the C–H···N hydrogen bonds toward the pyrazine ring nitrogen atom. These links were of substantial importance in the crystal structures of the most elastic materials, while in the structures of almost inelastic crystals they were notably longer and less influential.

Against this backdrop, we hypothesized that if we strengthen the weakest link in the crystal structure, we will consequently be able to enhance the elastic performance of our crystals. Moreover, changes in the mechanical output could be further achieved by altering the influence of the new link, the C–H···N hydrogen bond, in the crystal structure.

To strengthen the weakest link in the crystal structure, we opted for a cocrystal synthesis, as this synthetic method proved efficient in fine-tuning a range of physical properties via possessing a precise control over the supramolecular assembly.^22^ Since this method does not involve breaking or making new chemical bonds but solely relies on the formation of a targeted supramolecular link,^23^ it emerged as an ideal method to improve the targeted connection in the crystal structure of our initial low-performance material. Successful strengthening of the targeted link, through carefully selected appropriate cocrystallizing agents, should consequently result in the intended enhancement of the elastic behavior of our materials.

To test the applicability of our approach, we opted for 1,4-dicyanobenzene (1,4-DCB, **A**) and 1,4-dinitrobenzene (1,4-DNB, **B**) as small and symmetric cocrystallizing agents that, similarly to the starting pyrazine-based ligand, comprise a single aromatic ring. Thus, the least possible disturbance in the crystal structure of the parent coordination polymer (CP) was ensured, while the availability of the hydrogen atoms to strengthen the targeted supramolecular link was secured through the presence of effective electron-withdrawing functionalities in their vicinity (Figure 1).^24^ To assess the efficacy of the approach, the least elastic material from the family of variably elastic crystals, namely [CdI_2_(I-pz)_2_]_*n*_ (**1**; I-pz = iodopyrazine), was selected.^9^

**Figure 1:**
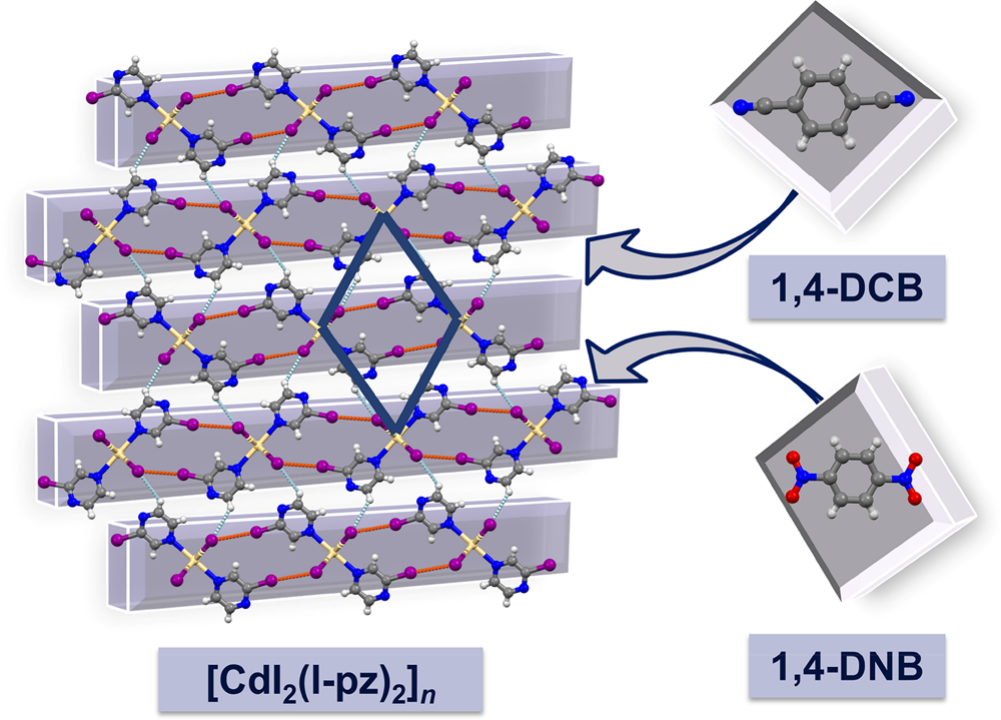
Parent coordination polymer (CP), [CdI_2_(I-pz)_2_]_*n*_ (**1**), with the crystal structure orthogonal to the elongation of the crystal. Bending faces are indicated as dark blue lines (rhombohedral shape), and a pale blue background shadowing indicates 2-D layered crystal packing with white regions comprising the least influential intermolecular interactions (only interactions shorter than the sum of the vdW radii are indicated), the weakest links in the crystal strucutre. hydrogen bonds indicated by light blue dashed lines), the weakest links in the crystal structure. Strengthening the weakest links via incorporating small organic cocrystallizing agents, 1,4-dicyanobenzene (1,4-DCB, **A**) and 1,4-dinitrobenzene (1,4-DNB, Stregnthening the weakest links via incorporating small organic cocrystallizing agents, 1,4-dicyanobenzene (1,4-DCB, **A**) and 1,4-dinitrobnzene (1,4-DNB, **B**), in the crystal structure of the parent CP.

The cocrystal synthesis was carried out via solvent-assisted grinding, using conditions modified from previously reported procedures^25^ (the formation of a new phase was confirmed by PXRD; see the Supporting Information), and the synthetic procedure was followed by a recrystallization of the phase-pure ground product from small amounts of methanol (**1:A**) and acetonitrile (**1:B**). Solvent evaporation yielded crystals of the required morphology (i.e., needle-like crystals) as well as quality for both the determination of crystal structures and testing mechanical performances (for details see the Supporting Information).

A crystal structure determination confirmed the delivery of the two targeted bicomponent materials, **1:A** and **1:B**, isostructural in the *P*2/*c* space group. Furthermore, it revealed that the newly introduced components (1,4-DCB, **A**; 1,4-DNB, **B**) did not essentially impair the structure of the parent CP (**1**) but rather were implemented between the 2-D polymeric layers (all now comprising CPs only in a parallel orientation), via the intended C–H···N links (Figure 2, bottom; Table S3). Moreover, in both cases (**1:A** and **1:B**) a substantially shorter and consequently stronger link than in the parent CP (**1**) was realized (Table 1), which accomplished our first goal, strengthening the weakest link in the parent CP’s structure.

**Table 1:** Normalized Hydrogen-Bonding Distances (Å) for **1**, **1:A**, and **1:B**^a^

	**1**	**1:A**	**1:B**
*R*_HA_(C–H···N)	1.10	0.94	0.97

a Normalized hydrogen-bonded distance, *R*, calculated according to Lommerse et al.^26^*R*_HA_ = *d*(H···A)/(*r*_H_ + *r*_A_), where *r*_H_ and *r*_A_ correspond to the van der Waals radii of hydrogen and the acceptor atoms, respectively (H, 1.20 Å; N, 1.55 Å).

**Figure 2:**
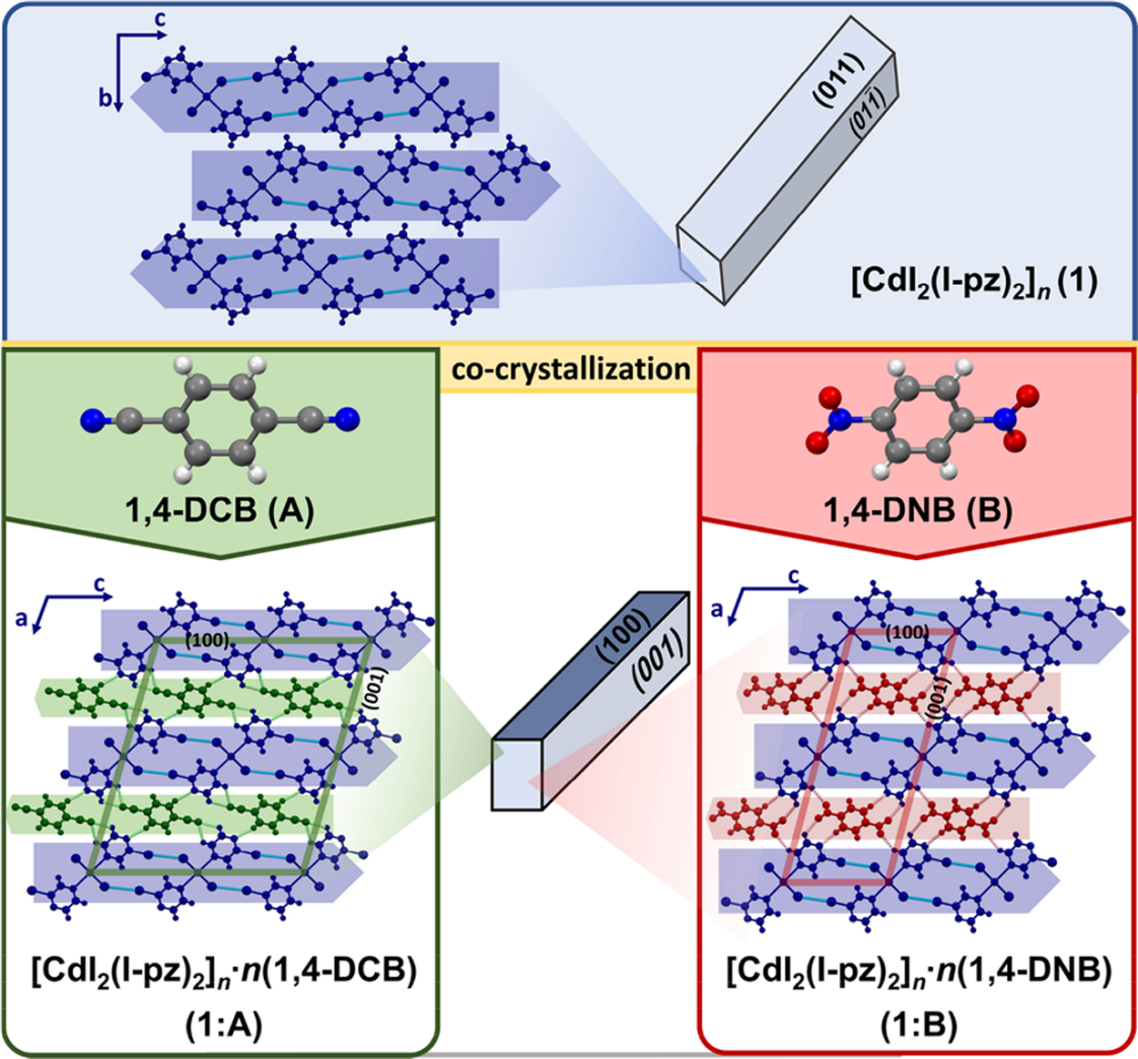
Crystal packing viewed down the direction of the elongation of crystals themselves together with the crystal morphologies: the parent coordination polymer [CdI_2_(I-pz)_2_]_*n*_ (**1**) (top) and modified materials **1:A** (bottom, left) and **1:B** (bottom, right).

An inspection of the crystal morphologies of **1:A** and **1:B** prior to testing their adaptability to an external mechanical force revealed a substantial morphology change with respect to the starting material **1**. While the parent CP yielded acicular crystals with equally developed two pairs of bending faces (Figure 2, top), **1:A** and **1:B** presented crystals with two different sets of bending faces that resemble highly elongated plate-like crystals more closely than the crystal morphology of the initial material itself (Figure 2, bottom). Moreover, even a notable distinction in morphologies of the two materials, **1:A** and **1:B**, emerged upon a detailed examination of their crystals; for **1:A**, two bending faces were only slightly different, while for **1:B** the difference was pronounced (Figure 3). All that in turn enabled us to examine the crystals’ performances (**1:A** and **1:B**) upon the application of force on both bending faces.

**Figure 3:**
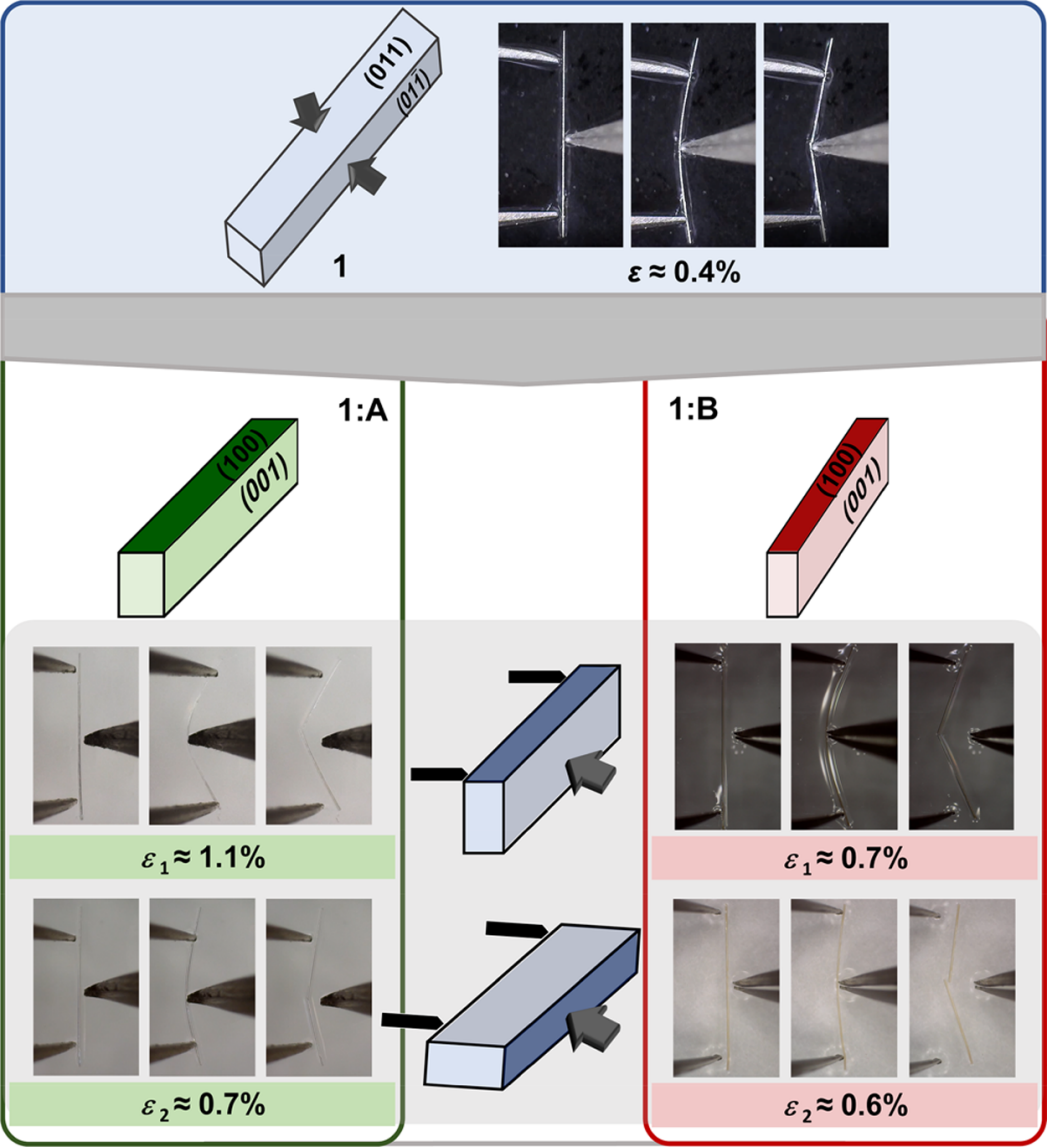
Parent coordination polymer (**1**) yielding crystals with equally developed crystal faces and presenting 2-D isotropic elastic bending, ε = 0.4% (top). Crystals of **1:A** (bottom, left) and **1:B** (bottom, right) presenting two pairs of bending faces, (100)/(100) and (001)/(001). The difference in the dimensions of (100)/(100) and (001)/(001) was relatively small for **1:A**, while it was pronounced for **1:B**. Both **1:A** and **1:B** presented 2-D anisotropic elastic adaptability to mechanical force, being more elastic when they were bent over the smaller bending faces, (100)/(100) (ε_1_: 1.1% **1:A**, 0.7% **1:B**), than when they were bent over the larger faces, (001)/(001) (ε_2_: 0.7% **1:A**, 0.6% **1:B**).

To examine the adaptability, elongated crystals of superb quality were selected and placed on glass slides coated with small amounts of Paratone oil to help handle the crystals, and a modified three-point bending procedure was utilized to examine their performances while they were exposed to an external mechanical force (viz. crystals were supported by two metal supporters from one side while the force was applied by moving the actuator in regular increments and in a controlled manner from the opposite side; Scheme S1).

Both **1:A** and **1:B** were expectedly elastic, but in contrast to the parent material **1**, **1:A** and **1:B** presented two elastic behaviors that were dependent on the direction of the force application and distinct from each other solely with respect to their extents (Figure 3). Both materials were more elastic when they were bent over the smaller face (i.e., the force applied to the crystal faces of larger dimensions, ε_1_; Figures S9 and S12, movie 1, and movie 3) and less elastic when they were bent over the larger faces (ε_2_; Figures S10 and S13, movie 2, and movie 4). Thus, **1:A** and **1:B** joined a small family of 2-D anisotropically flexible materials: materials that are flexible in two directions perpendicular to the axial direction of the crystals themselves but display two flexibility extents.^15^

The elasticities of **1:A** and **1:B** were further assessed using the Euler–Bernoulli equation^27^ to yield the bending strain values, ε, thus allowing us to evaluate the efficacy of our approach (for more details see the Supporting Information). The retrieved bending strain values, ε_1_ and ε_2_ (ε_1_, application of the force to the faces of larger dimensions, (001)/(001); ε_2_, application of the force to the faces of smaller dimensions, (100)/(100)), revealed that the intended enhancement of the elastic adaptability of **1:A** and **1:B** was indeed achieved. Both **1:A** and **1:B** presented notably improved elastic performances regardless of the bending face to which the force was applied, with **1:A** being more elastic than **1:B** (ε = 0.4% (**1**) → ε_1_ = 1.1%, ε_2_ = 0.7% (**1:A**); ε = 0.4% (**1**) → ε_1_ = 0.7%, ε_2_ = 0.6% (**1:B**); Tables S5–S8). With this, the delivery of modified materials **1:A** and **1:B** that clearly presented enhanced elasticity, the efficacy of our approach was undoubtedly confirmed.

Rationalization of the mechanical outcome against the backdrop of the geometrical features of the newly introduced C–H···N link revealed a surprisingly good correlation of the difference in the elasticities of **1:A** and **1:B** and shortening of the link; the shorter the link, the more enhanced the elasticity of the material (*R*_HA_(**1:A**) < *R*_HA_(**1:B**) → ε_1_, ε_2_ (**1:A**) > ε_1_, ε_2_ (**1:B**); Table 1). Moreover, since the link was not lying in any specific orientation with respect to either of the bending faces (i.e., directed along or parallel) but rather was inclined to those, it apparently influenced the flexibilities of crystals over both bending faces, which consequently resulted in enhanced elasticities of the crystals in both dimensions orthogonal to the elongation of the crystals themselves (ε_1_, ε_2_).

In addition to successfully enhancing the elastic flexibility of the two carefully designed materials which subsequently demonstrated the validity and efficacy of our approach, the two materials **1:A** and **1:B** offered several other insights into understanding the impact and influence of various structural features on the mechanical elasticity of crystalline materials.

First, despite being isostructural, the two materials yielded crystals of notably different morphologies. While the bending faces of **1:B** were substantially different in their dimensions, those of **1:A** were more similar (Figure 3), most probably as a consequence of an additional hydrogen bond of the C–H···O type (*R*_HA_ = 0.92; Table S3) present in **1:B** and absent in **1:A**.

Moreover, the two materials also displayed distinct differences in elastic extents for bending over the two pairs of bending faces, (100)/(100) and (001)/(001). Crystals of **1:B** were almost equally elastic when they were bent over the two bending faces (**1:B**: ε_1_ = 0.7%, ε_2_ = 0.6%) despite their substantially different dimensions. In contrast, crystals of **1:A** presented a substantial difference in elasticity despite a smaller difference in the bending face dimensions (**1:A**: ε_1_ = 1.1%, ε_2_ = 0.7%). This nicely depicted that the elastic performances of a crystal do not simply correlate with the dimensions of a particular crystal face but rather are a consequence of the inherent structural anisotropy of the material itself.

Furthermore, while supramolecular interactions have already proven essential for the delivery of a range of different mechanical outputs and their extents,^9,14,28,29^ here the arrangement and orientation of molecular features with respect to a particular bending face might also have an impact on engendering crystals with a difference in elasticities of both **1:A** and **1:B**. While individual molecules (1,4-DCB and 1,4-DNB) elongate along the [001] direction and are anchored in the crystal structure solely by supramolecular links, the individual polymeric units of the CP are oriented along the [1̅01] direction (i.e., the direction of attachment of the iodopyrazine ligands) and are mutually connected via covalent bonds into a polymeric chain that propagates along the [010] direction. Thus, the CP as the largest and the most rigid constituent of the overall material’s structure is most likely the critical feature for enabling crystal flexibility, being more prone to absorb and compensate for slight structural changes^9,30,31^ in the direction orthogonal to the orientation of individual polymeric units than along the direction of polymeric unit attachment. what consequently, might reflect on the difference in mechanically induced elasticity along two distinct directions. An extensive analysis of these features is currently underway in our group.

Herein, the two isostructural materials, **1:A** and **1:B**, in addition to proving the validity and efficacy of our approach, also offered invaluable insights into the structural background of a specific mechanical output. While identifying the critical link in the structure of a parent material proved crucial for rational design of materials with improved mechanical performances, cocrystallization emerged as a practical trajectory for their delivery.

With this carefully designed materials **1:A** and **1:B**, we have straightforwardly presented that suboptimal mechanical properties of crystalline metal-containing solids can be intentionally improved, which in turn provides a promising avenue for the improving flexibility of many high-value crystalline materials with low initial elastic performances, thus consequently making them readily available for application in a variety of advanced technologies.

## Supporting Information Available

Synthetic procedures for the preparation of **1:A** and **1:B**, PXRD results for **1:A** and **1:B**, SCXRD results for **1:A** and **1:B**, TG/DSC analysis for **1:A** and **1:B**, and crystal bending experiments for **1:A** and **1:B** (PDF)
**1:A** applying the force on the larger face (AVI)
**1:A** applying the force on the smaller face (AVI)
**1:B** applying the force on the larger face (AVI)
**1:B** applying the force on the smaller face (AVI)

## Supplementary Material

cg2c01397_si_001.pdf

cg2c01397_si_002.avi

cg2c01397_si_003.avi

cg2c01397_si_004.avi

cg2c01397_si_005.avi
