# Supplementary material for: Tailoring Enhanced Elasticity of Crystalline Coordination Polymers
Source: Cryst Growth Des. 2023 Feb 13;23(3):1318–22. doi: 10.1021/acs.cgd.2c01397 (PMC9983303; doi:10.1021/acs.cgd.2c01397)
Supplement: Supplementary file 1 — cg2c01397_si_001.pdf [file cg2c01397_si_001.pdf]

Tailoring Enhanced Elasticity of Crystalline Coordination Polymers

Ozana Mišura,[a] Mateja Pisačić,[a] Mladen Borovina,[a] and Marijana Đaković[a]*

[a] *Department of Chemistry, Faculty of Science, University of Zagreb, Zagreb, Croatia*

Supplementary information

Table of Contents

1. Co-crystal synthesis and growing crystals 3
2. Powder X-ray crystallography 5
3. Single crystal X-ray crystallography 8
4. Thermal analysis 12
5. Crystal bending experiments 13
6. References 20

1. Co-crystal synthesis and growing crystals

All solvents and reagents were purchased from commercial suppliers and used without further purification. Parent 1-D coordination polymer (CP), $[CdI_2(I\text{-}pz)_2]_n$ (**1**), was prepared following the literature procedure,[1] while the co-crystals synthesis was carried out *via* solvent-assisted grinding whereby the parent 1-D CP was combined with two organic co-formers, 1,4-dicyanobenzene, 1,4-DCB (**A**) and 1,4-dinitrobenzene, 1,4-DNB (**B**).

Co-crystal synthesis

The targeted co-crystals were synthesized using conditions modified from reported procedures for the preparation of Cd(II) coordination polymers.[2]

A reaction mixture of **1** (1 eq.) and organic co-former (1 eq.) was placed in a 10 mL stainless steel jar with 40 µL of ethanol and 2 stainless steel balls (7 mm in diameter) and ground for 60 minutes at 25 Hz frequency. Grinding was carried out in the Retsch MM200 ball mill. Once the solvent had evaporated the ground mixture was analyzed by powder X-ray diffraction (PXRD). The formation of a co-crystalline substance was confirmed by a comparison of experimental PXRD traces of the resulting grinding products with those of the starting substances.

$[CdI_2(I\text{-}pz)_2]_n\cdot n$(1,4-DCB), 1:A. Used: $[CdI_2(I\text{-}pz)_2]_n$ (100.3 mg; 0.129 mmol) and 1,4-DCB (17.1 mg; 0.133 mmol). A comparison of PXRD traces revealed the presence of new diffraction maxima in the PXRD pattern of the resulting mixture, **1:A** (Figure S3).

$[CdI_2(I\text{-}pz)_2]_n\cdot n$(1,4-DNB), 1:B. Used: $[CdI_2(I\text{-}pz)_2]_n$ (99.7 mg; 0.128 mmol) and 1,4-DNB (21.4 mg; 0.127 mmol). A comparison of PXRD traces revealed the presence of new diffraction maxima in the PXRD pattern of the resulting mixture, **1:B** (Figure S4).

Growing crystals

The resulting grinding product was dissolved in methanol (**1:A**) or acetonitrile (**1:B**) at room temperature, and the solution was placed in a test tube, closed by parafilm (with a few holes to allow slow evaporation), and left undisturbed at ambient conditions to obtain single crystals suitable for testing mechanical adaptability to external mechanical force. Within a week, colorless (**1**:**A**) and yellowish (**1**:**B**) needle-like crystals of the required quality were harvested.

The phase purity of the final products was examined by analysis of the powder X-ray diffraction (PXRD) patterns; the PXRD of the bulk crystals was performed and compared with the calculated powder pattern of the co-crystal, **1**:**A** and **1**:**B**.

$[CdI_2(I\text{-}pz)_2]_n\cdot n$(1,4-DCB), 1:A. Used: $[CdI_2(I\text{-}pz)_2]_n\cdot n$(1,4-DCB) in 1 mL of methanol. The powder X-ray diffraction pattern (bulk sample) was consistent with the pattern calculated from the single-crystal data (Figure S5).

[CdI$_2$(I-pz)$_2$]$_n$·*n*(1,4-DNB), **1:B.** Used: [CdI$_2$(I-pz)$_2$]$_n$·*n*(1,4-DNB) in 1 mL of acetonitrile. The powder X-ray diffraction pattern (bulk sample) was consistent with the pattern calculated from the single-crystal data (Figure S6).

2. Powder X-ray crystallography

Polycrystalline samples were finely ground and placed on a silicon plate for powder X-ray diffraction (PXRD) experiments which were performed on a Malvern Panalytical Aeris powder diffractometer in the Bragg-Brentano geometry with PIXcel^{1D} detector under an applied voltage of 40 kV and current of 15.0 mA. The radiation used was Cu*K*α (*l* = 1,5406 Å), and all patterns were collected at room temperature, from

4° to 60° (2*θ*) and with a step size of 0.02°. The PXRD data were collected for the resulting grinding products, **1:A** (Figure S1) and **1:B** (Figure S2), as well as for the bulk re-crystallization samples, **1:A** (Figure S3) and **1:B** (Figure S4).

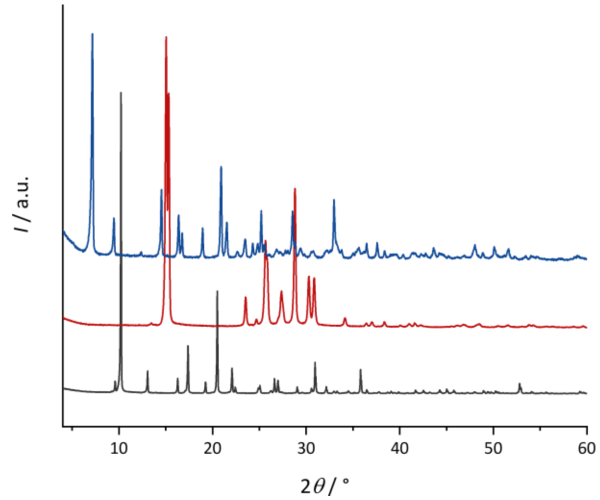

Figure S1. Experimental PXRD traces of starting compounds, $[CdI_2(I\text{-}pz)_2]_n$ (**1**, **black**) and 1,4-DCB (**A**, **red**), and the resulting grinding product (**1:A**, **blue**).

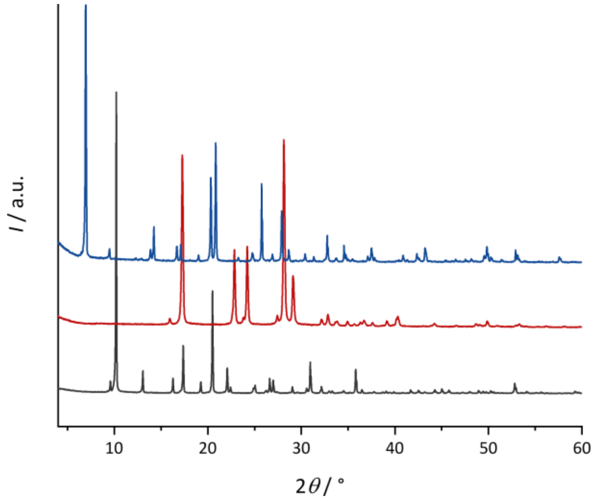

Figure S2. Experimental PXRD traces of starting compounds, $[CdI_2(I\text{-}pz)_2]_n$ (**1**, **black**) and 1,4-DNB (**B**, **red**), and the resulting grinding product (**1:B**, **blue**).

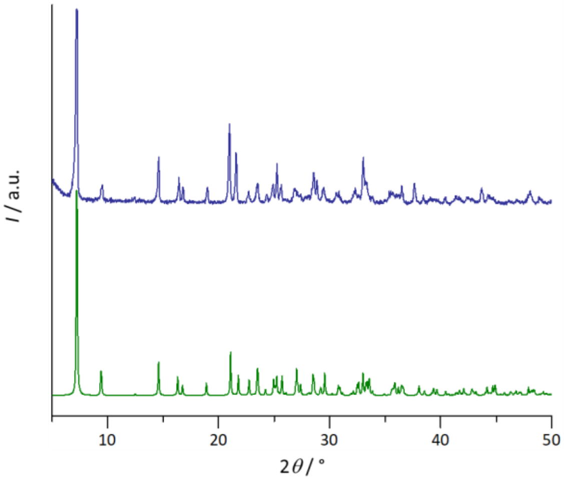

Figure S3. Calculated (**green**) and experimental (**blue**) PXRD traces of $[CdI_2(I\text{-}pz)_2]_n{\cdot}n$(1,4-DCB) (**1:A**).

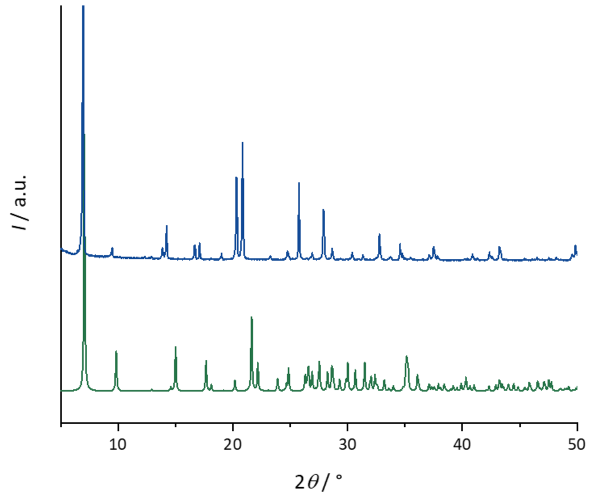

Figure S4. Calculated (**green**) and experimental (**blue**) PXRD traces of $[CdI_2(I\text{-}pz)_2]_n{\cdot}n$(1,4-DNB) (**1:B**).

3. Single crystal X-ray crystallography

Suitable crystals for single-crystal X-ray experiments were isolated from the mother liquor and mounted in a random orientation on a glass fiber. Data collections were carried out on an XtaLAB Synergy-S Dualflex diffractometer with PhotonJet (Mo) microfocus X-ray source and HyPix-6000HE hybrid photon counting (HPC) X-ray area detector and applying the CrysAlisPro Software system[3] at 295(2) K. Data reduction, including absorption correction, was done by CrysAlisPro program. The structures were solved by SHELXT program.[4] The coordinates and the anisotropic thermal parameters for all non-hydrogen atoms were refined by full-matrix least-squares methods based on F^2 using the SHELXL program. The hydrogen atoms were generated geometrically using the riding model with the isotropic factor set at $1.2U_{eq}$.

Graphical work has been performed by Mercury 2021.3.0 software (version 4.3.1).[5] The thermal ellipsoids were drawn at the 50% probability level. General and crystal data with the summary of intensity data collection and structure refinement for compounds **1:A** and **1:B** are given in Table S1.

CCDC 2191195–2191196 contain the supplementary crystallographic data for this paper.

Table S1. Crystal data and details of the structure determination for **1:A** and **1:B.**

**Compound**	**1:A**	**1:B**
Formula moiety	$(C_8H_6CdI_4N_4)(C_8H_4N_2)$	$(C_8H_6CdI_4N_4)(C_6H_4N_2O_4)$
Empirical formula	$C_{16}H_{10}CdI_4N_6$	$C_{14}H_{10}CdI_4N_6O_4$
M_r	906.30	946.28
Color and habit	colorless, needle	yellowish, needle-like
Crystal system, space group	Monoclinic, *P*2/c (No. 13)	Monoclinic, *P*2/c (No. 13)
Crystal dimensions (mm^3)	0.3 x 0.04 x 0.03	0.19 x 0.06 x 0.04
a (Å)	12.8614(2)	13.2624(6)
b (Å)	4.15590(10)	4.18350(10)
c (Å)	22.0543(4)	21.5031(9)
α (Å)	90	90
β (Å)	105.469(2)	105.216(4)
γ (Å)	90	90
V ($Å^3$)	1136.11(4)	1151.24(8)
Z	2	2
ρ_{calc} (g cm^{-3})	2.649	2.730
μ (Mo-K_α) (mm^{-1})	6.409	6.344
F(000)	816	856
T temperature (K)	295(2)	295(2)
λ radiation wavelength (Å)	Mo K_α	Mo K_α
θ range for data collection (°)	$1.92 \leq \theta \leq 31.00$	$2.18 \leq \theta \leq 29.99$
h, k, l range	$-18 \leq h \leq 18$<br>$-6 \leq k \leq 6$<br>$-31 \leq l \leq 31$	$-18 \leq h \leq 18$<br>$-5 \leq k \leq 5$<br>$-30 \leq l \leq 30$
Scan type	ω	ω
No. measured reflections	82370	16674
No. independent reflections (R_{int})	3605	3345
No. observed reflections, $I \geq 2\sigma(I)$	3271	2688
No. refined parameters	123	132
R, wR [$I \geq 2\sigma(I)$]	0.0191, 0.0477	0.0280, 0.0633
R, wR [all data]	0.0222, 0.0489	0.0381, 0.0671
Goodness of fit on F^2, S	1.018	1.011
Max., min. electron density (e $Å^{-3}$)	− 0.545, 1.077	− 0.696, 0.667
CCDC number	2191195	2191196

Table S2. Selected bond distances (Å) and angles (˚) for **1:A** and **1:B**.

	**1:A**	**1:B**
Bond distances		
Cd1–N1	2.448(2)	2.448(3)
Cd1–I1	2.951(1)	2.971(1)
Cd1–I1^{i}	2.945(1)	2.931(1)
Bond angles		
Cd1–I1–Cd1^{i}	89.65(1)	90.30(1)
I1–Cd1–I1^{ii}	90.24(1)	88.93(2)
I1–Cd1–I1^{iii}	89.64(1)	90.30(1)
I1^{iii}–Cd1–I1^{iv}	90.48(1)	90.48(2)
N1–Cd1–I1	89.70(5)	89.55(7)
N1–Cd1–I1^{ii}	90.22(5)	88.82(7)
N1–Cd1–I1^{iii}	89.78(5)	91.17(7)
N1–Cd1–I1^{iv}	90.30(5)	90.44(7)

Symmetry codes: (*i*) *x*, *y* – 1, *z*; (*ii*) –*x*, *y*, –*z* + 3/2; (*iii*) *x*, *y* + 1, *z*; (*iv*) –*x*, *y* + 1, –*z* + 3/2.

Table S3. Hydrogen bond distances and angles for **1:A** and **1:B**.

Compound	C–H···A	*d*(C···A) / Å	*d*(H···A) / Å	∠(C–H···A) / °	R_{HA}*
	C7–H7···N2^{i}	3.503(4)	2.59	167	0.94
**1:A**	C3–H3···N3^{ii}	3.452(5)	2.88	121	1.05
	C4–H4···N3^{ii}	3.455(5)	2.90	119	1.05
	C7–H7···N2^{i}	3.526(6)	2.63	162	0.97
**1:B**	C3–H3···O2^{iii}	3.380(9)	2.76	125	1.01
	C4–H4···O1	3.138(7)	2.50	126	0.92

Symmetry codes: (i) *x*, –*y* + 1, *z* +1/2; (*ii*) *x*, *y* + 1, *z*; (iii) –*x* + 1, *y* + 1, –*z* + 3/2;

*Normalized value *R*, defined according to Lommerse et al.[6] $R_{HA} = d(H\cdots A) / (r_H + r_A)$, where r_H and r_A correspond to van der Waals radii of hydrogen and acceptor atoms (H 1.20 Å, N 1.55 Å, O 1.52 Å).

Table S4. Halogen bond distances (Å) and angles (˚) for **1:A** and **1:B**.

Compound	C–X···A	*d*(X···A) / Å	∠(C–X···A) / °	R_{XA}*
**1:A**	C2–I2···I1^{i}	3.717(1)	173.09(6)	0.94
**1:B**	C2–I2···I1^{i}	3.697(1)	175.19(10)	0.93

Symmetry codes: (*i*) –*x*, –*y*, –*z* + 1;

*Normalized value *R*, defined according to Lommerse et al.[5] $R_{XA} = d(X\cdots A) / (r_X + r_A)$, where r_X and r_A correspond to van der Waals radii of halogen bond donor and acceptor atoms (I 1.98 Å).

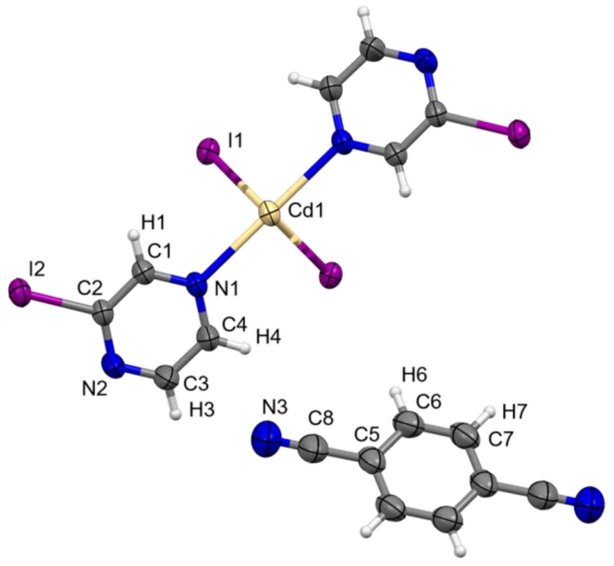

(a)

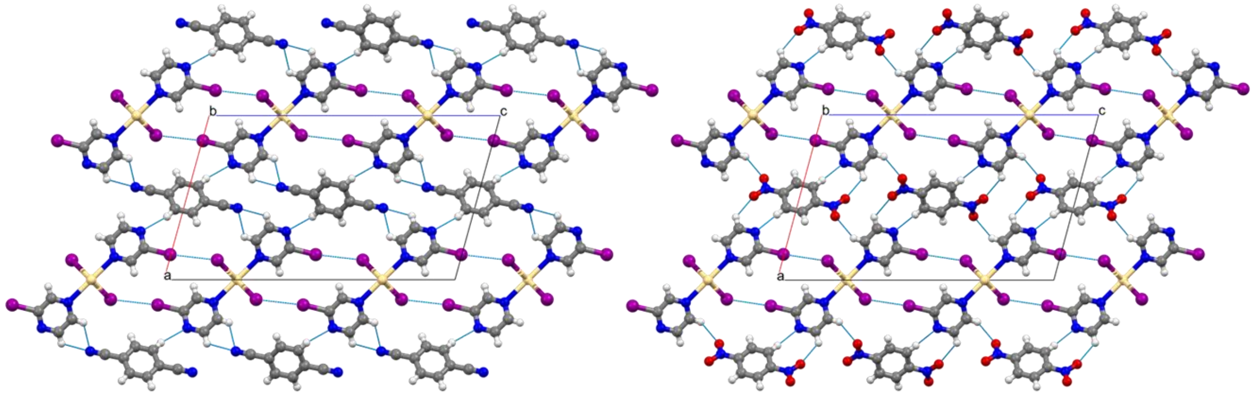

(b)

Figure S5. ORTEP-style plots of **1:A** (a, left) and **1:B** (a, right) with a partial labeling scheme. Thermal ellipsoids are drawn at 50% probability level at 295(2) K. Crystal packing of **1:A** (b, left) and **1:B** (b, right) with the unit cell axes with indicated hydrogen and halogen bonds as dashed blue lines.

4. Thermal analysis

Thermogravimetric analysis was performed using a simultaneous TGA-DTA analyzer Mettler-Toledo TGA/DSC 3+. The powder samples (**1:A**, **1:B**) were placed in alumina pans (70 μL) and heated in flowing nitrogen (50 mL min^{-1}) from room temperature up to 600 °C at a rate of 10 °C min^{-1}. Data collection and analysis were performed using the program package STARe Software v.16.30.[7]

The mass losses that occurred in the first steps of both compounds (**1:A**: 59.41%; **1:B**: 60.05%) correspond to the loss of two I-pz ligands and one molecule of the co-formers (**1:A**: 1,4-DCB; **1:B**: 1,4-DNB). Temperatures of the decompositions (**1:A**: 165 °C; **1:B**: 185 °C) reflect the impact of different supramolecular interactions present in **1:A** and **1:B** on the stability of their crystals.

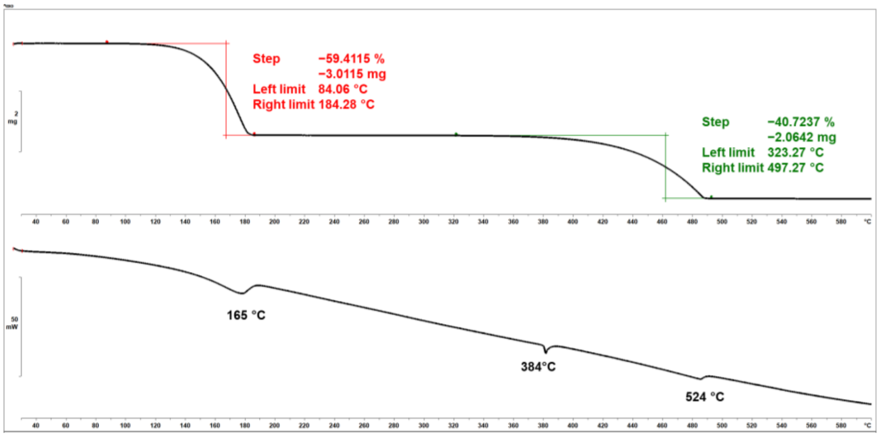

Figure S6. TGA (top) and DSC (bottom) curves of **1**:**A**.

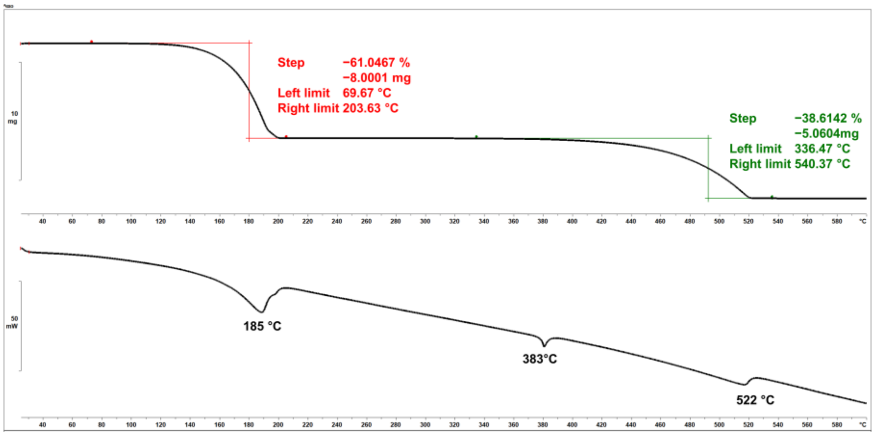

Figure S7. TGA (top) and DSC (bottom) curves of **1**:**B**.

5. Crystal bending experiments

For conducting experiments on crystal bending, needle-like crystals were isolated from the mother liquor and placed on a glass slide with a few drops of paratone oil added. A modified three-point bending procedure was employed using a pair of metal tweezers; the crystal was anchored from one side in two points and thus supported, while the mechanical force was applied with a metal needle from the opposite side (perpendicular to the elongation of the crystal) in a controlled manner by motorized moving the metal needle in regular increments (Δd = 30 μm) and at constant velocity (v = 100 μm/s).

Bending was performed upon both pairs of prominent crystal faces, i.e. the force was applied to the crystal face of smaller dimension, $(100)/(\bar{1}00)$, and to the crystal face of larger dimension, $(001)/(00\bar{1})$. In case the crystals are not initially positioned on the smaller crystal face, upon application of the force they flip on the crystal face of a smaller dimension, then flip back upon crystal fracture (movies 1–4). All bending experiments were carried out using a Dino-Lite Edge Digital Microscope (model AM4815ZT), and the recordings were taken and processed using DinoCapture 2.0 software (version 1.5.40.B).

The parameters for calculating the bending strain value (***ε***): the distance between two points (***L***) where the crystal was anchored and the maximal displacement ($\boldsymbol{h}_{\mathbf{max}}$) of the crystal at the moment of maximal curvature, were measured just before the crystal fracture (Figs. S9, S10, S12, S13). The thickness of the crystal (***t***) was measured at the straight crystals' parts, while the radius of the curvature (***R***) was calculated from the geometric construction that approximated the crystal curvature by a circle (Scheme S1, Equation S1, Equation S2). The elastic flexible response was then quantified using the Euler-Bernoulli equation[8] (considering pure bending without shear component, Equation S3).

$$R^2 = \left(\frac{L}{2}\right)^2 + (R - h_{\mathrm{max}})^2 \qquad \text{Equation S1}$$

$$R = \frac{1}{2}\frac{\left(\frac{L}{2}\right)^2 + {h_{\mathrm{max}}}^2}{h_{\mathrm{max}}} \qquad \text{Equation S2}$$

$$\varepsilon\ (\%) = \frac{\frac{t}{2}}{R} \cdot 100 \qquad \text{Equation S3}$$

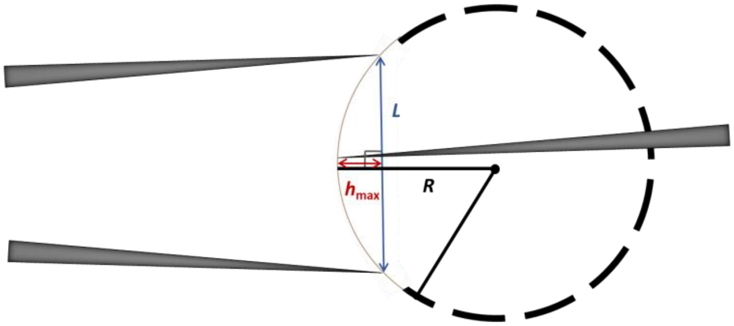

Scheme 1. A three-point bending experiment in schematic representation: the distance between two supporters (blue double arrow, *L*); maximal displacement (red double arrow, h_{max}). The maximal curvature of the bent crystal was approximated by a circle of radius *R* (black line, *R*).

Mechanical bending of crystals of [CdI_2(I-pz)$_2$]$_n$·n(1,4-DCB), 1:A

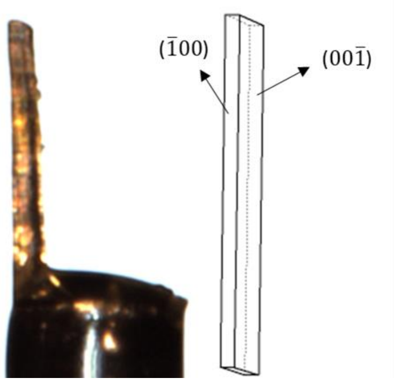

Figure S8. Crystal morphology of [CdI_2(I-pz)$_2$]$_n$·n(1,4-DCB) (**1:A**) with two pairs of prominent crystal faces indicated by corresponding Miller indices $(100)/(\bar{1}00)$ and $(001)/(00\bar{1})$.

Table S5. Geometrical parameters used to calculate the bending strain (ε) for **1:A** upon application of the mechanical force to the larger crystal faces, $(001)/(00\bar{1})$. Length (L) and maximal displacement (h_{max}) of the selected crystals were measured at the point of maximal curvature, while thickness (t) was measured at the straight crystals' parts.

Crystal sample	t_1 / mm	t_2 / mm	t_3 / mm	$\bar{t}$ / mm	L / mm	h_{max} / mm	R / mm	ε / %
1	0.028	0.031	0.029	0.030	2.326	0.489	1.627	**0.90**
2	0.024	0.027	0.027	0.026	1.172	0.191	0.994	**1.31**
3	0.034	0.028	0.032	0.031	1.002	0.107	1.226	**1.28**
4	0.028	0.028	0.032	0.029	0.979	0.111	1.135	**1.29**
5	0.037	0.034	0.037	0.036	1.541	0.157	1.969	**0.91**
6	0.046	0.041	0.046	0.044	1.094	0.058	2.608	**0.85**
7	0.042	0.041	0.040	0.041	2.136	0.236	2.535	**0.81**
8	0.046	0.044	0.046	0.045	1.094	0.058	2.608	**0.87**
9	0.037	0.037	0.035	0.036	1.058	0.075	1.903	**0.95**
10	0.030	0.031	0.033	0.031	1.494	0.276	1.149	**1.36**
							**$\bar{\varepsilon}$ / %**	**1.1 ± 0.2**

Table S6. Geometrical parameters used to calculate the bending strain (ε) for **1:A** upon application of the mechanical force to the smaller crystal faces, (100)/($\bar{1}$00). Length (L) and maximal displacement (h_{max}) of the selected crystals were measured at the point of maximal curvature, while thickness (t) was measured at the straight crystals' parts.

Crystal sample	t_1 / mm	t_2 / mm	t_3 / mm	$\bar{t}$ / mm	L / mm	h_{max} / mm	R / mm	ε / %
1	0.046	0.046	0.046	0.046	2.210	0.180	3.482	**0.66**
2	0.042	0.046	0.046	0.045	4.111	0.655	3.553	**0.63**
3	0.041	0.044	0.042	0.042	2.481	0.244	3.275	**0.65**
4	0.042	0.038	0.041	0.040	1.971	0.155	3.210	**0.63**
5	0.037	0.034	0.037	0.036	2.077	0.218	2.583	**0.70**
6	0.013	0.013	0.009	0.012	0.470	0.034	0.829	**0.70**
7	0.046	0.046	0.047	0.046	2.863	0.332	3.252	**0.71**
8	0.032	0.028	0.027	0.029	1.895	0.227	2.091	**0.69**
9	0.025	0.025	0.026	0.025	1.335	0.116	1.979	**0.64**
							$\bar{\varepsilon}$ / %	**0.67 ± 0.03**

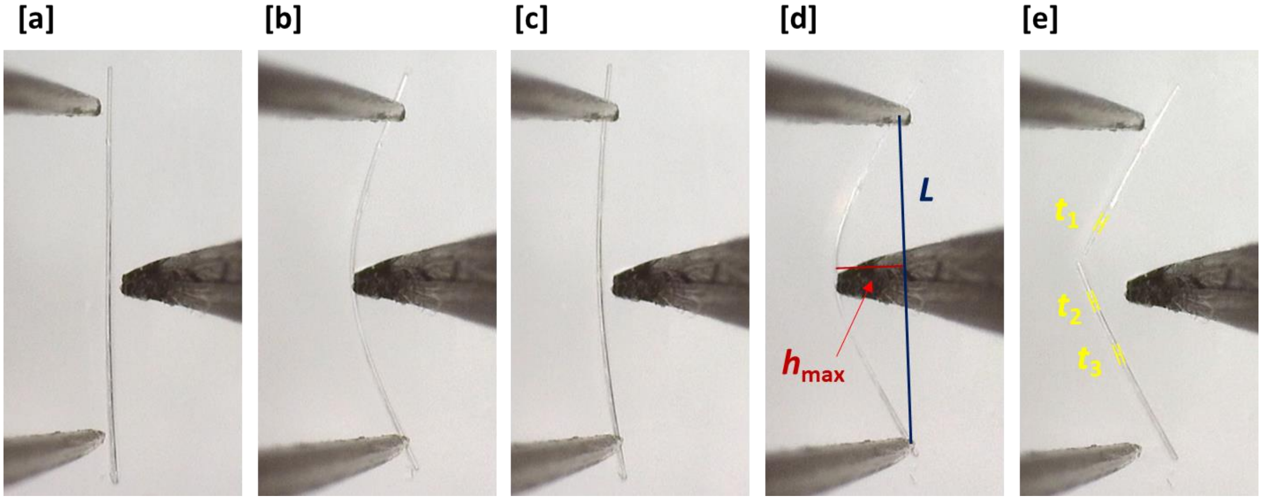

Figure S9. Elastic bending of **1:A** (images **a**–**e** magnified 50 times) upon application of the mechanical force to the face of larger dimension, (001)/(00$\bar{1}$) (movie 1). Elastic bending of the crystal followed by relaxation (**a**–**c**); crystal breaks as bent over the critical radius (**d**–**e**). Geometrical parameters, length, ***L*** (L = 2.326 mm), and maximal displacement, $\boldsymbol{h_{max}}$ (h_{max} = 0.489 mm), were measured at the point of maximal crystal curvature (**d**), and were followed by the measurement of crystal thickness, ***t*** (t_1 = 0.028 mm, t_2 = 0.031 mm, t_3 = 0.029 mm) after crystal breaking (**e**). The bending strain was calculated (ε = 1.1%).

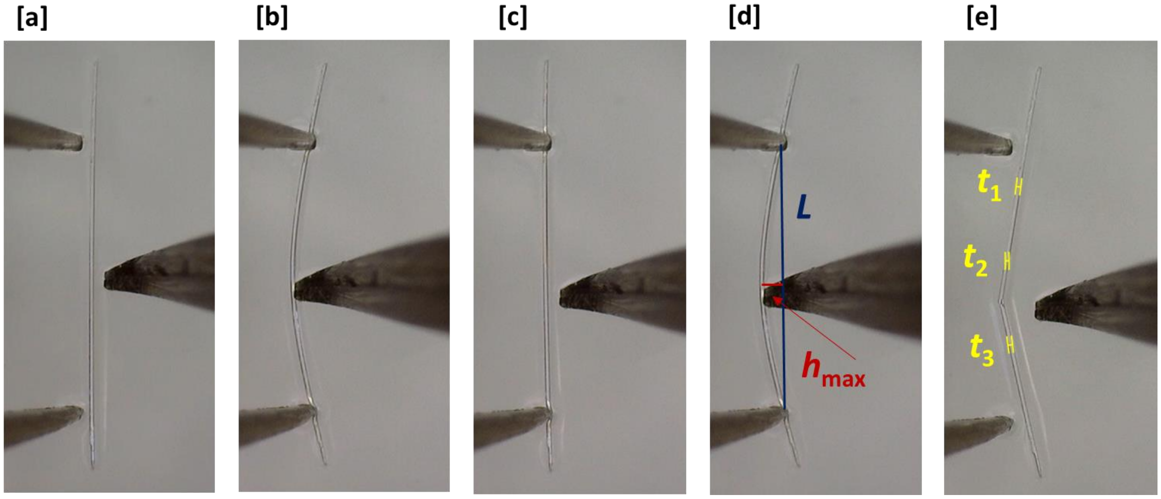

Figure S10. Elastic bending of **1:A** (images **a**–**e** magnified 50 times) upon application of the mechanical force applied to the face of smaller dimension, $(100)/(\bar{1}00)$ (movie 2). Slight elastic bending of the crystal followed by relaxation (**a**–**c**); crystal breaks as bent over the critical radius (**d**–**e**). Geometrical parameters length, ***L*** (L = 2.210 mm), and maximal displacement, $\boldsymbol{h}_{\mathbf{max}}$ (h_{max} = 0.180 mm), were measured at the point of maximal crystal curvature (**d**), and were followed by the measurement of crystal thickness, ***t*** (t_1 = 0.046 mm, t_2 = 0.046 mm, t_3 = 0.046 mm) after crystal breaking (**e**). The bending strain was calculated (ε = 0.67%).

Mechanical bending of crystals of $[CdI_2(I\text{-}pz)_2]_n{\cdot}n$(1,4-DNB), 1:B

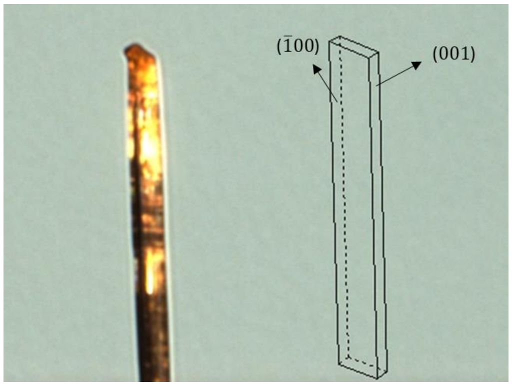

Figure S11. Crystal morphology of $[CdI_2(I\text{-}pz)_2]_n{\cdot}n$(1,4-DNB) (**1:B**) with two pairs of the prominent faces indicated by corresponding Miller indices $(100)/(\bar{1}00)$ and $(001)/(00\bar{1})$.

Table S7. Geometrical parameters used to calculate the bending strain (ε) of **1:B** upon application of the mechanical force to the crystal face of larger dimension, $(001)/(00\bar{1})$. Length (L) and maximal displacement (h_{max}) of the selected crystals were measured at the point of maximal curvature, while thickness (t) was measured at the straight crystals' parts.

Crystal sample	t_1 / mm	t_2 / mm	t_3 / mm	$\bar{t}$ / mm	L / mm	h_{max} / mm	R / mm	ε / %
1	0.078	0.078	0.079	0.078	5.563	0.741	5.591	**0.70**
2	0.056	0.058	0.056	0.057	3.572	0.431	3.916	**0.72**
3	0.052	0.052	0.054	0.053	4.672	0.921	3.423	**0.77**
4	0.119	0.117	0.123	0.120	4.999	0.368	8.672	**0.69**
5	0.061	0.064	0.058	0.061	3.841	0.553	3.611	**0.84**
6	0.079	0.078	0.082	0.080	5.012	0.586	5.651	**0.70**
7	0.078	0.079	0.082	0.080	3.313	0.228	6.132	**0.65**
8	0.051	0.051	0.048	0.050	3.954	0.522	4.005	**0.62**
9	0.036	0.037	0.037	0.037	2.889	0.466	2.472	**0.74**
10	0.055	0.055	0.051	0.054	3.814	0.553	3.565	**0.75**
							$\bar{\varepsilon}$ / %	**0.72 ± 0.06**

Table S8. Geometrical parameters used to calculate the bending strain (ε) for **1:B** upon application of the mechanical force to the crystal face of smaller dimension, $(100)/(\bar{1}00)$. Length (L) and maximal displacement (h_{max}) of the selected crystals were measured at the point of maximal curvature of the crystal, while thickness (t) was measured at the straight crystals' parts.

Crystal sample	t_1 / mm	t_2 / mm	t_3 / mm	$\bar{t}$ / mm	L / mm	h_{max} / mm	R / mm	ε / %
1	0.033	0.028	0.033	0.031	2.775	0.362	2.840	**0.55**
2	0.059	0.061	0.066	0.062	3.964	0.414	4.951	**0.63**
3	0.055	0.051	0.051	0.052	1.992	0.120	4.193	**0.62**
4	0.042	0.042	0.046	0.043	1.699	0.103	3.555	**0.61**
5	0.099	0.105	0.101	0.102	3.064	0.124	9.526	**0.53**
6	0.087	0.092	0.092	0.090	3.084	0.142	8.443	**0.53**
7	0.087	0.092	0.088	0.089	2.821	0.119	8.419	**0.53**
8	0.092	0.091	0.088	0.090	3.070	0.146	8.142	**0.55**
9	0.032	0.032	0.031	0.032	2.784	0.372	2.790	**0.57**
							$\bar{\varepsilon}$ / %	**0.57 ± 0.04**

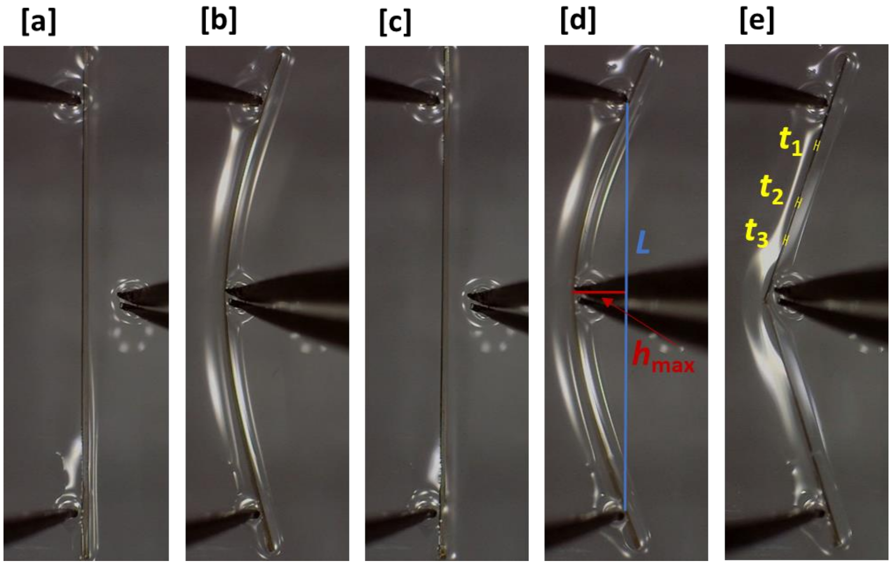

Figure S12. Elastic bending of **1:A** (images **a**–**e** magnified 50 times) upon application of the mechanical force to the face of larger dimension, $(001)/(00\bar{1})$ (movie 3). Elastic bending of the crystal followed by relaxation (**a**–**c**); crystal breaks as bent over the critical radius (**d**–**e**). Geometrical parameters, length, ***L*** (L = 5.563 mm), and maximal displacement, $\boldsymbol{h}_{\mathbf{max}}$ (h_{max} = 0.741 mm), were measured at the point of maximal crystal curvature (**d**), and were followed by the measurement of crystal thickness, ***t*** (t_1 = 0.078 mm, t_2 = 0.078 mm, t_3 = 0.079 mm) after crystal breaking (**e**). The bending strain was calculated (ε = 0.72%).

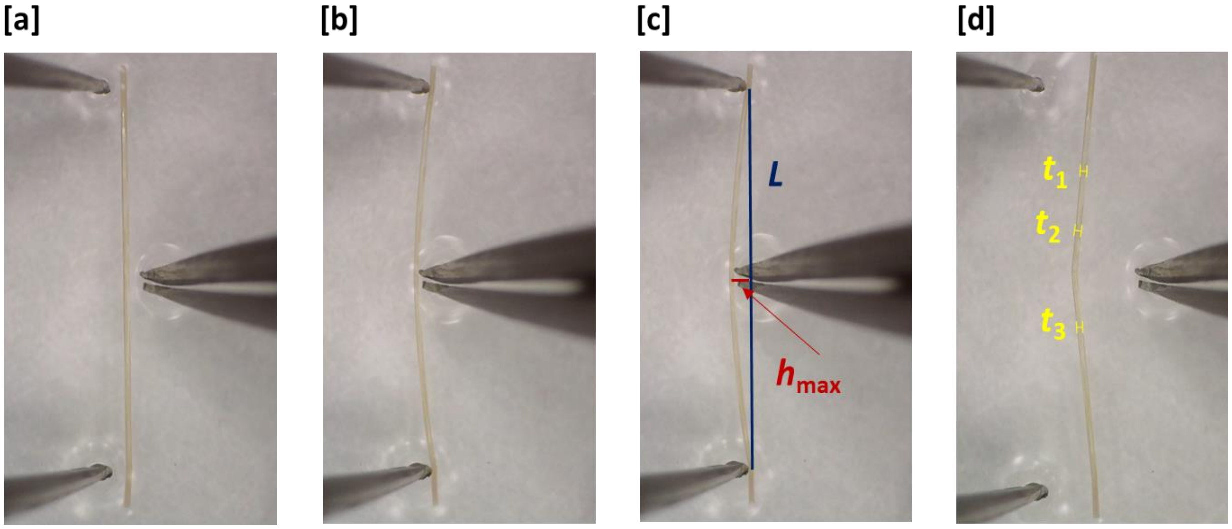

Figure S13. Elastic bending of **1:B** (images **a**–**d** magnified 50 times) upon application of the mechanical force to the face of smaller dimension, $(100)/(\bar{1}00)$ (movie 4). Slight elastic bending of the crystal (**a**–**b**); crystal breaks as bent over the critical radius (**c**–**d**). Geometrical parameters length, ***L*** (L = 2.775 mm), and maximal displacement, $\boldsymbol{h}_{\mathbf{max}}$ (h_{max} = 0.362 mm), were measured at the point of maximal crystal curvature (**c**), and were followed by the measurement of crystal thickness, ***t*** (t_1 = 0.033 mm, t_2 = 0.028 mm, t_3 = 0.033 mm) after crystal breaking (**d**). The bending strain was calculated (ε = 0.57%).

6. References

1. Đaković, M.; Borovina, M.; Pisačić, M.; Aakeroy, C. B.; Soldin, Ž.; Kukovec B.-M.; Kodrin, I. Mechanically Responsive Crystalline Coordination Polymers with Controllable Elasticity. *Angew. Chem. Int. Ed.* **2018**, *57*, 14801–14805.
2. Đaković, M.; Soldin, Ž.; Kukovec B.-M.; Kodrin, I.; Aakeröy, C. B.; Baus, N.; Rinkovec, T. Building inorganic supramolecular architectures using principles adopted from the organic solid state. *IUCrJ* **2018**, *5*, 13–21.
3. CrysAlisPRO, Oxford Diffraction/Agilent Technologies UK Ltd, Yarnton, England.
4. Sheldrick, G. M. SHELXT-Integrated space-group and crystal-structure determination. *Acta Crystallogr*. **2015**, *A*71, 3–8.
5. Macrae, C. F.; Sovago, I.; Cottrell, S. J.; Galek, P. T. A.; McCabe, P.; Pidcock, E.; Platings, M.; Shields, G. P.; Stevens, J. S.; Towler, M.; Wood, P. A. Mercury 4.0: from visualization to analysis, design and prediction. *J. Appl. Cryst.* **2020**, *53*, 226–235.
6. Lommerse, J. P. M.; Stone, A. J.; Taylor, R.; Allen, F. H. The Nature and Geometry of Intermolecular Interactions between Halogens and Oxygen or Nitrogen. *J. Am. Chem. Soc.*, 1996, **118**, 3108–3116.
7. STAReSoftware 16.20, MettlerToledoGmbH, **2006**.
8. Timoshenko, S. *Strength of materials*, D. Van Nostrand Company, New York, 1940.
